# Feed-Forward Propagation of Temporal and Rate Information between Cortical Populations during Coherent Activation in Engineered *In Vitro* Networks

**DOI:** 10.3389/fncir.2016.00032

**Published:** 2016-04-22

**Authors:** Thomas B. DeMarse, Liangbin Pan, Sankaraleengam Alagapan, Gregory J. Brewer, Bruce C. Wheeler

**Affiliations:** ^1^J. Crayton Pruitt Family Department of Biomedical Engineering, University of FloridaGainesville, FL, USA; ^2^Department of Pediatric Neurology, University of FloridaGainesville, FL, USA; ^3^Department of Bioengineering, University of CaliforniaIrvine, CA, USA; ^4^Department of Bioengineering, University of CaliforniaSan Diego, CA, USA

**Keywords:** information transmission, feed-forward networks, multielectrode array, cortical networks, neural populations, neural transmission, neuronal assembly, synchrony

## Abstract

Transient propagation of information across neuronal assembles is thought to underlie many cognitive processes. However, the nature of the neural code that is embedded within these transmissions remains uncertain. Much of our understanding of how information is transmitted among these assemblies has been derived from computational models. While these models have been instrumental in understanding these processes they often make simplifying assumptions about the biophysical properties of neurons that may influence the nature and properties expressed. To address this issue we created an *in vitro* analog of a feed-forward network composed of two small populations (also referred to as assemblies or layers) of living dissociated rat cortical neurons. The populations were separated by, and communicated through, a microelectromechanical systems (MEMS) device containing a strip of microscale tunnels. Delayed culturing of one population in the first layer followed by the second a few days later induced the unidirectional growth of axons through the microtunnels resulting in a primarily feed-forward communication between these two small neural populations. In this study we systematically manipulated the number of tunnels that connected each layer and hence, the number of axons providing communication between those populations. We then assess the effect of reducing the number of tunnels has upon the properties of between-layer communication capacity and fidelity of neural transmission among spike trains transmitted across and within layers. We show evidence based on Victor-Purpura’s and van Rossum’s spike train similarity metrics supporting the presence of both rate and temporal information embedded within these transmissions whose fidelity increased during communication both between and within layers when the number of tunnels are increased. We also provide evidence reinforcing the role of synchronized activity upon transmission fidelity during the spontaneous synchronized network burst events that propagated between layers and highlight the potential applications of these MEMs devices as a tool for further investigation of structure and functional dynamics among neural populations.

## Introduction

Transiently active neuronal assemblies are thought to underlie many operations within the brain and provide the basis for a number of complex cognitive processes including recall, thinking, planning and decision-making (Buzsáki, 2010). Although neural assemblies propagate information in the form of spiking activity, the nature of the neural code underlying that communication is still a matter of debate (Barlow, 1972; Gray, 1994; König et al., 1996; Shadlen and Newsome, 1998; Shadlen and Movshon, 1999; Van Rullen and Thorpe, 2001). According to one view, the emergence of synchronous correlated neuronal firing is the salient feature necessary for the transmission of temporally precise signals between assemblies (McCormick and Prince, 1987; Gray and Singer, 1989; Murthy and Fetz, 1992; Abeles et al., 1993; Hatsopoulos et al., 1998; Prut et al., 1998; Baker et al., 1999; Diesmann et al., 1999; Câteau and Fukai, 2001; Gewaltig et al., 2001; Reyes, 2003; Fries, 2005) and in some cases does so with high temporal precision (Abeles et al., 1993; Mainen and Sejnowski, 1995; Nowak et al., 1997; Riehle et al., 1997; Prut et al., 1998). A complementary view emphasizes a neural code based on the modulation of the rate of firing in which the number, not the timing of spiking is important (Barlow, 1972, 2009; Shadlen and Newsome, 1994, 1998; van Rossum et al., 2002). In fact, there have been suggestions that synchrony may actually be detrimental to successful transmission under some circumstances for the transmission of for example, rate information (Barlow, 1972, 2009; Shadlen and Newsome, 1998; Mazurek and Shadlen, 2002). However, current views now suggest that this apparent dichotomy may simply be a matter of time scale at which information is assessed, and that both codes likely co-exist simultaneously, leading a number of investigators to begin to reconcile these seemingly disparate views into a unified understanding of these properties and their relationship to memory and cognition (e.g., Kumar et al., 2010; Ainsworth et al., 2012; Taillefumier and Magnasco, 2013).

**Figure 1:**
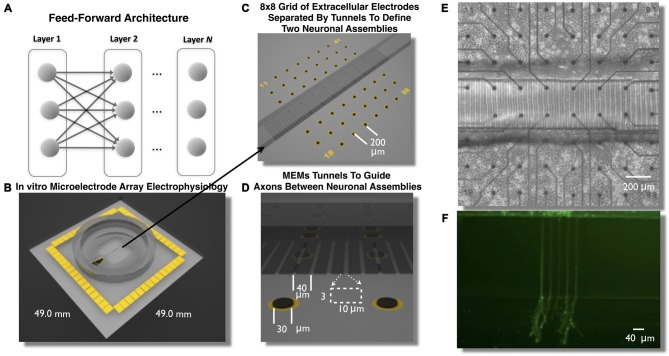
**Feed-Forward network architecture and *in vitro* microelectromechanical systems (MEMS) device for the study of neural transmission in living cortical networks.** While there are many unique network topologies within the brain the feed-forward network, illustrated in **(A)**, is commonly used as a generic model to study the properties of neural transmission of information. We created a living analog of a two-layer feed-forward network to address this issue using a polydimethylsiloxane (PDMS) MEMs device shown in **(B,C)**. Each device was located over an array of 60 extracellular microelectrodes **(C–E)** to measure propagation of neural activity (action potentials) and assess the properties of neural transmission of information between neurons embedded within layers of living cortical neurons *in vitro*. Each layer of cortical neurons was separated by micro-scale tunnels **(C–E)** that permitted axonal growth between layers but prohibited soma from entering the tunnels detailed in **(D)**. Through timed sequential plating neurons in Layer I extend neurites into Layer II (shown in **F**, calcien image after 3 days in vitro) we achieved directional feed-forward connectivity in a two layer feed-forward network. We then study the transmission fidelity of information encoded in spike trains during the propagation of network activity across each layer.

Any neural code should serve at least four key functions. It should be reproducible and provide a reliable stimulus representation. It should be interpretable, can transform information, and be capable of transmission or propagation (Perkel and Bullock, 1968). In this article we will focus on the last of these properties, transmission, which has received surprisingly little experimental attention (Kumar et al., 2010). In fact, much of our understanding of the properties of the neural code during transmission have been derived from modeling studies that reduce complex biological systems to a limited set of algorithms and architectures (Diesmann et al., 1999; Câteau and Fukai, 2001; Kistler and Gerstner, 2002; van Rossum et al., 2002; Litvak et al., 2003; Vogels and Abbott, 2005; Kumar et al., 2008, 2010) that may alter the expression of rate, temporal, or other codes. Perhaps one of the most commonly studied network architectures has been the feed-forward network topology illustrated in Figure 1A. This topology is often constructed as a simple but useful structural analogy thought to capture important features of information transmission between the different brain regions (Felleman and Van Essen, 1991; Young, 1993; Kikuchi et al., 2010; Modha and Singh, 2010). Unfortunately *in vivo* testing of hypotheses or direct manipulation of factors related to neuronal transmission among assemblies has been experimentally difficult likely due to the inherent complexity and limited accessibility of the brain. To address the question, an ideal preparation might consist of only a few well-defined layers of connected small neuronal populations that are combined with simultaneous parallel electrophysiological measurements of network activity.

A classic solution to the problem of accessibility has been to employ *in vitro* neuronal cell culture. In this model neural tissue is harvested from the brain, dissociated, cultured, and studied in detail *in vitro*. These cultured networks form functional, spontaneously active assemblies within a few days of plating. They also offer extraordinary accessibility, and have been widely used to study the fundamentals of neural computation including plasticity at the individual neuron (e.g., Debanne and Poo, 2010) to network scales (e.g., Dranias et al., 2013), for pharmacology (e.g., Morefield et al., 2000), biosensors (e.g., Stenger et al., 2001; Selinger et al., 2004; Scarlatos et al., 2008), and recently shown by our group to spontaneously engage in neuronal oscillations at theta and gamma frequencies (Leondopulos et al., 2012), that are now suspected of underlying attention (Jensen et al., 2007), memory formation (Axmacher et al., 2006), and whose abnormalities may underlie a number of psychiatric disorders (Le Van Quyen et al., 2006; Uhlhaas and Singer, 2006). These gamma oscillations may also play a prominent role in the encoding and transmission of information within and between neuronal assemblies (Fries et al., 2007; Lisman and Jensen, 2013). However until recently this living *in vitro* model of *in vivo* computation lacked the capability to constrain the neuronal architecture with sufficient detail to re-construct these networks *in vitro* with sufficient functional relations between neuronal assemblies that can easily be manipulated and studied. Fortunately a variety of microelectromechanical systems (MEMS) based technologies have now matured enabling the “engineering” of neuronal growth and creation of arbitrary neuronal architectures. These technologies have led to a number of advances toward more refined models of *in vivo* neuronal architectures reconstituted within *in vitro* systems (Wheeler and Brewer, 2010; Nam and Wheeler, 2011).

In this article we describe the creation of a living feed-forward network composed of two layers each containing a small neuronal population of rat cortical neurons and each layer is interconnected using a micro-tunnel device illustrated in Figures 1B–F. Each micro-tunnel device was positioned over a 60-electrode microelectrode array (MEA; Figures 1C,E) to enable detailed temporal and spatial measurement of neural activity within each layer and any propagation of activity through the tunnels (magnified in Figure 1D) via a series of two electrodes within select tunnels into the second layer. We first systematically manipulate the number of tunnels that connect each layer using 2, 5, 10, 15, and 51 tunnels (Pan et al., 2011, 2015). This effectively limits the number of axons that interconnect each neural population via the tunnels and therefore, the amount of communication possible between each layer. In our recent report we have shown that even this rather simple manipulation resulted in a significant effect upon the coupling strength (i.e., number of functional connections) between layers and the likelihood and delay in which electrical stimulation of a single electrode in Layer I evoked bursts of neural activity that successfully propagated into Layer II (Pan et al., 2015). In this article we now investigate the spontaneous network dynamics that develop within this system and delineate and quantify the communication fidelity during transmission of spike trains as they are propagated between neurons embedded within two distinct neuronal populations in Layer I to Layer II. We systematically increase the physical connectivity between each layer by increasing the number of tunnels and hence, potential communication capacity between these layers and resultant increase in transmission fidelity at course rate based and more precise temporal scales.

## Materials and Methods

### Design of *In Vitro* Feed-Forward Neuronal Networks

A variety of technologies now exist to effectively engineer neuronal structure *in vitro*. For example, stamping or other deposition methodologies of neuronal growth factors or adhesion promoters have for many years been used to create simple grid networks (e.g., Corey et al., 1996; Branch et al., 2000; Kam et al., 2001; Kumar et al., 2010), investigate cell morphogenesis (Théry, 2010), or for the study of spinal injury and repair (Taylor et al., 2005, 2009). Alternatively the natural tendency of neurons to follow structural features including ridges (Curtis and Wilkinson, 1997), pillars (Dowell-Mesfin et al., 2004) or application of microfluidics to guide axonal growth (Morin et al., 2006) have also been exploited toward the study of structural-functional relationships of biological neural networks. A key limitation of each of these techniques, however, is that they do not provide a means to control the *directionality* of connections, a property needed to establish and study feed-forward connectivity during communication between neuronal populations.

Fortunately this technical hurdle has now been overcome using a variety of methods including electrical fields (McCaig et al., 2002), microstamped features (Stenger et al., 1998; Natarajan et al., 2013) or polydimethylsiloxane (PDMS) structures (Feinerman et al., 2008; Dworak and Wheeler, 2009; Pan et al., 2011, 2015; Brewer et al., 2013), and other surface topology to guide growth cones (Hattori et al., 2010) and in combination with neuronal cell culture, offer the possibility of creating defined *in vitro* neuronal populations with feed-forward connectivity. In fact, microtunnel-like devices have had an extensive history (Campenot, 1977) and have recently been adapted toward the study of neural systems including research using dissociated neuronal cultures that form small self-organized and spontaneously active networks composed of a few thousand or up to tens of thousands of neurons (e.g., Taylor et al., 2003, 2005, 2009; Pearce et al., 2005; Berdondini et al., 2006; Morin et al., 2006; Ravula et al., 2007; Feinerman et al., 2008; Liu et al., 2008a; Dworak and Wheeler, 2009; Park et al., 2009; Yang et al., 2009; Shi et al., 2010; Taylor and Jeon, 2010; Wieringa et al., 2010; Kanagasabapathi et al., 2011, 2012, 2013; Pan et al., 2011; Peyrin et al., 2011; Biffi et al., 2012; Bisio et al., 2014; Sung et al., 2014; Tang-Schomer et al., 2014) and organotypic brain slice culture (Berdichevsky et al., 2010, 2012).

To create these cultures embryonic day 18 (E18) cells including neurons and glia are dissociated into individual cells from rat cortical hemispheres (Brain Bits, LLC) and plated to a final density of 1500 cells/mm^2^ into one of the two chambers illustrated in Figures 1B–E. The first chamber in which cells are placed will become the first layer (Layer I) of the feed forward network. Over the following days cortical neurons in Layer I rapidly extend growth cones to neighboring cells within this layer. These growth cones eventually enter into the microtunnels connecting each layer (Pan et al., 2011) and rapidly traverse the length of a 400 μm tunnels reaching the second chamber in about 10 days. This second chamber is then plated with cells to form the second layer (Layer II) of the feed-forward network. The combined effect of the occlusion of tunnels by axons extending from the first layer (Layer I) and the likely presence of guidance cues from soma and growth cones within Layer II result in a system of two distinct neuronal assemblies unidirectionally connected into a functional two-layer feed forward network architecture (Pan et al., 2011, 2015). To assess the properties of neural transmission both within and between layers, each layer was cultured over an array of extracellular electrodes (8 × 8 grid of 30 μm electrodes, 200 μm spacing) that measured spiking activity from changes in extracellular voltage. Each MEA was aligned such that each layer contained 22 electrodes. The two center rows of electrodes were aligned within and along the length of the tunnels (Figures 1C,D).

### Manipulating the Degree of Connectivity (Communication Pathways) Between Layers and its Effects on Neural Transmission

In this study we manipulated the number of tunnels connecting each layer in an attempt to directly manipulate the amount of communication and potential information exchanged between layers. In our prior report we showed that increasing the number of tunnels resulted in more functional connections and faster propagation of synchronous activity between layers during electrically evoked bursts of synchronous activity (Pan et al., 2015). Here we hypothesize that the number of tunnels may represent a rough approximation for the eventual number of axonal projections that reach Layer II. This in turn should affect the number of potential communication paths and therefore the amount of information that can be carried across these tunnels to the neural population in the opposite chamber. Our prediction is that by increasing the number of communication pathways, we will increase the fidelity of information during its transmission between neurons from Layer I to Layer II. To accomplish this, five groups of cultures were created, each with increasing number of tunnels connecting each layer. In this case, PDMS tunnel devices were created with 2 tunnels (Group 2T, *n* = 4 cultures), 5 tunnels (Group 5T, *n* = 5), 10 tunnels (Group 10T, *n* = 5), 15 tunnels (Group 15T, *n* = 4), and 51 tunnels (Group 51T, *n* = 4) between each neuronal population. Neuronal cell cultures were derived from two independent cell culture preparations that spanned a three-month period.

### Microtunnel Device Fabrication

A detailed description of the fabrication process of the microtunnels can be found in our previous articles (Dworak and Wheeler, 2009; Pan et al., 2011, 2015). Briefly, SU-8 2002 (Microchem, Inc.) was spun onto a 4-inch silicon wafer at a thickness of 3 μm, baked at 95°C, exposed with the first mask for the tunnels, baked at 95°C again and developed in SU-8 developer. The surrounding chamber structure was created by spin-coating SU-8 2050 (Microchem, Inc.) at a nominal thickness of 120 μm and then baked at 95°C. The second mask was aligned with alignment marks within the first SU-8 film and exposed, baked again and developed. And at this point the mold that was created from this process was ready for casting PDMS microtunnel devices. PDMS (Sylguard 184, Dow Corning) was poured on the wafer slowly and allowed to spread over the whole wafer, which was then put on a hotplate for curing (2 h at 70°C). The cured PDMS layer was peeled off the wafer for use. Two chambers on either side of the tunnel were punched out with a steel biopsy punch (5 mm, compressed along one dimension to form an oval with a straight edge along the tunnels). A third smaller hole was created (2 mm) over the reference electrode. Each microtunnel was 3 μm tall, 10 μm wide and 400 μm long and spaced 40 μm (center-to-center); and each chamber’s dimension was 3 mm × 5 mm.

### Cell Culture

Prior to the cell culture, the surface of each MEA was plasma cleaned for 10 min and then coated overnight with poly-D-lysine (PDL) solution (100 μg/ml, diluted in borate buffer at pH of 8.5). The following day the MEAs with microtunnel devices were rinsed three times by sterilized de-ionized (DI) water and then dried. A microtunnel device was aligned with an MEA by using a customized aligner (XYZ stage with three angular rotations under a microscope) in such a way that the two rows of electrodes lie within the tunnels. Pressure is applied to create a seal between the PDMS device and an MEA. Neurobasal^™^/B27/GlutaMAX^™^ (Invitrogen, Inc.) media was then added in each culture chamber and incubated at 5% CO_2_ and 37°C for several hours to allow media to penetrate into the tunnels before plating cells.

Embryonic E18 rat cortical hemispheres purchased from BrainBits LLC (Springfield, IL, USA) were dissociated according to the vendor’s protocol. Cell harvesting procedures were approved by the University of Florida and SIUSM animal care committees. MEAs were removed from the incubator, the media was aspirated from the first chamber and 20 μl of cell suspension (1.5 × 10^6^ cells/ml) was added to form Layer I of the feed-forward network. Each MEA was placed in the incubator for 10 min to permit cell attachment followed by the addition of 300 μl of media providing a reservoir large enough to withstand evaporation losses and ambient CO_2_ without significant pH change. MEA cultures were then returned to the incubator. Half of the media was exchanged every 2 days. Seven days later, the media was removed from the second chamber and cells plated to form Layer II. The ages of the cultures (days *in vitro* or DIV) in this report are all referred to the date of the initial plating of Layer I.

### Electrophysiology

Recordings of neural activity were conducted from 21 to 26 DIV using a commercial multichannel signal amplifier (MEA 1060BC, Multi Channel Systems, Inc gain 1200×, sampling rate 25 KHz, Bandwidth 1 Hz–10 KHz). Raw electrophysiological signals were filtered (300 Hz–3 KHz) and putative action potentials (spikes) were detected online using MEABench (Wagenaar et al., 2005) as positive or negative excursions beyond 5.0× estimated root-mean-square (RMS) noise gathered for each electrode during the first 15 s of each recording. Electrodes that produced spikes with rates less than 0.01 spikes per second are typically due to noise alone and were dropped from further analysis.

Spikes were then sorted using the surrounding ±1 ms of each spike’s waveform using the first three components from a principal components analysis (PCA) followed by unsupervised k-means based on the KlustaKwik method (Harris et al., 2000). There were no significant differences between groups in the average number of neurons per electrode (*m* = 1.37 ± 0.04, *p* > 0.06) but Layer I was slightly elevated relative to Layer II (1.46 ± 0.13 vs. 1.28 ± 0.11, Layer I and II respectively, *p* < 0.05).

### Spike Train Analysis

#### Burst Detection

Virtually every neuronal cell type that is cultured with sufficient density will develop into a spontaneously active network of neurons producing action potentials concomitantly with increases in the number of synapses (Muramoto et al., 1993) and maturation of phenotype (Stephens et al., 2012) as early as the first few days after plating. The overall pattern of firing gradually evolves during the formation of these networks with the initial appearance of isolated action potentials within the first few days that gradually form small isolated but interconnected subpopulations that oscillate in the form of brief localized bursting. After 7–10 DIV, these isolated network oscillations begin to coalesce into bursts of activity that engage the entire network and whose firing patterns (“motifs”) evolve over the course of the following weeks (van Pelt et al., 2004, 2005; Stegenga et al., 2008; Downes et al., 2012; Gritsun et al., 2012; Pu et al., 2013). *In vitro* multielectrode arrays have been used to measure network bursting in a variety of cell types including cells from cortex (Kamioka et al., 1996; Voigt et al., 1997; Jimbo et al., 2000; Segev et al., 2002; Tateno et al., 2002; Raichman et al., 2006; Pasquale et al., 2008; Downes et al., 2012), hippocampus (Leinekugel et al., 2002; Cohen and Segal, 2011), superchiasmatic nucleus (Welsh et al., 1995; Welsh and Reppert, 1996; Herzog et al., 2004; Herzog, 2007; Klisch et al., 2006; Granados-Fuentes et al., 2012; Mazzoccoli et al., 2012), cerebellum (Kleinfeld et al., 1988; Blenkinsop and Lang, 2011), and spinal cord (Kleinfeld et al., 1988; Gross and Kowalski, 1999; Yvon et al., 2005; Stegenga et al., 2008; Czarnecki et al., 2009). Activity early in the maturation of these cultures is reminiscent of spatiotemporal patterns seen in developing networks *in vivo* (Ben-Ari, 2001) and at approximately 30 DIV, may loosely resemble activity *in vivo* (Huettner and Baughman, 1986; McCormick and Prince, 1987; Kamioka et al., 1996; Nakanishi and Kukita, 1998, 2000; Leondopulos et al., 2012).

Network bursts were detected separately within each layer using the summex method (Wagenaar et al., 2005). Briefly, spike trains for each electrode were searched individually for burstlets (sequences of at least four spikes with interspike intervals less than a threshold set to 25% of that electrodes inverse average spike rate). Any group of burstlets that overlapped in time and across channels was considered a burst. Minimum burst durations were enforced at 10 ms. Peak spike rates during each burst and time associated with that peak were estimated from a smoothed histogram of spike counts (5 ms Gaussian blur, 1 ms bins).

#### Measures of Functional Connectivity

There are now a number of examples where functional connectivity has been estimated from spike train information among *in vitro* networks recorded with MEAs (e.g., Bettencourt et al., 2007; Garofalo et al., 2009; Feldt et al., 2010; Kanagasabapathi et al., 2011; Downes et al., 2012; Maccione et al., 2012; Poli et al., 2014; Pirino et al., 2015) and among networks cultured within tunnels devices similar to those used here (Kanagasabapathi et al., 2012). To verify the directionality of neural transmission from Layer I to Layer II we computed standard cross-correlograms (CCs) constructed from spike trains produced by spike-sorted signals from axons measured within the tunnels. Any communication between Layer I and II must cross through the tunnels in the form of a propagation of a signal along the axons within those tunnels whose precise timing can be measured from electrode pairs along those tunnels presenting a unique opportunity to assess directionality of communication between layers. Moreover due to the confined space within the tunnels and resultant high resistance, the magnitude of spike signals measured at electrodes within tunnels is often on the order of several mV, far larger than 10–100 μV typical of the extracellular spikes measured by electrodes in the open field of the chambers (Dworak and Wheeler, 2009; Pan et al., 2011, 2014). In our system a subset of electrode pairs were aligned length-wise along each tunnel. Only a subset of electrodes could be aligned and exposed within the tunnels due to the 40 μm center-to-center tunnel spacing (cf. Figure 1D) vs. 200 μm electrode spacing. Because of this alignment the maximum number of electrodes available for recording in the tunnels was one pair (2 electrodes) for the 2T, one pair for the 5T, two pairs for the 10T, three pairs for 15T, and seven pairs of electrodes along tunnels for the 51T devices.

In addition, we computed a conditional form of Granger causality (CGC) based on spike train information derived from *in vitro* MEA recordings (Cadotte et al., 2008) and reports by other laboratories (Kispersky et al., 2011). CGC was computed along the tunnel electrodes embedded within each tunnel to provide further support for the desired directionality and also provide a quantitative measure of the strength of functional connectivity between neurons among the assemblies in Layer I and II. Originally developed within the economics community by Clive Granger (Granger, 1969), the idea can be traced back to Wiener (1956) in which for any two simultaneously recorded time series, one series can be called causal to another if incorporating past knowledge of the first time series permits more accurate prediction of the second. Granger (1969) formalized this notion in the context of linear regression and estimation of functional connectivity using Granger causal metrics. While this pairwise technique can alone be quite useful to unravel any interdependencies in a network and outline its functional structure, it can encounter significant limitations in more complex networks where the relationship between a pair of neurons (or signals recorded at electrodes) can be mediated by other neurons, which is a much more common scenario. A CGC metric was used to overcome some of these limitations in which the influence of any mediating connections are compensated for in each pairwise comparison (Ding et al., 2006; Chen et al., 2006). The analysis was conducted using the Matlab/C libraries for Granger causal analysis (Cui et al., 2008; Seth, 2010). Spike trains were constructed from neural activity recorded over a 2-min period at each active electrode (up to 59 electrodes). Spike times were binned at 1 ms, and smoothed with a 4 ms exponential filter, following a method that we have previously used for spike data from random cortical cultures measured using MEAs (Cadotte et al., 2008). Due to the large number pairwise electrode comparisons and hence the large number of potential false positives, false discovery rate corrections were performed based on the approach of Benjamini and Hochberg (1995).

### Spike Train Similarity Metrics

We wish to compare the fidelity with which information embedded within spike trains is transmitted between the two layers of each feed-forward network and more importantly, how that fidelity changes when the communication capacity is reduced by decreasing the number of tunnels. We also wish to quantify the degree to which that information is primarily composed of rate information and whether there is also any evidence for a temporal coding within spike trains transmitted between each layer. There are now a number of metrics that have been proposed that have been used to quantify and compare the similarity between spike trains including spike train dissimilarity metrics such as Victor-Purpura’s cost based measure (Victor and Purpura, 1996, 1997; Victor, 2005), van Rossum’s kernel based approach (van Rossum et al., 2002), measures based on mutual information (e.g., Bettencourt et al., 2007), or more recent parameter free proposals including the ISI-Distance (Kreuz et al., 2007, 2009) and spike-distance (Kreuz et al., 2011). Of these, two common measures are the Victor-Purpura and van Rossum metrics that have been applied to data obtained from a variety of neural systems to quantify variability (MacLeod et al., 1998; Kreiman et al., 2000; Reinagel and Reid, 2002; Grewe et al., 2003; Chichilnisky and Rieke, 2005; Warzecha et al., 2013), characterize temporal neural coding between single neurons (Victor and Purpura, 1997; Mechler et al., 1998; Machens et al., 2001; Reich et al., 2001; Di Lorenzo and Victor, 2003; Rosen et al., 2011), or neuronal pairs (Nelson, 2002; Aronov et al., 2003; Samonds and Bonds, 2004).

The spike train distance measure introduced by Victor and Purpura (Victor and Purpura, 1996, 1997) defines the distance (dissimilarity) between two spike trains as the “edit” distance or minimum cost, ***D_v_***, of transforming one spike train into the other following a series edit operations (insertion, deletion, or temporal shifting). While the operations of insertion and deletion have a fixed cost of 1, the cost of shifting a spike in time Δt is ***q***|Δ*t*|. The parameter, ***q***, adjusts the cost per unit time and therefore sets the relative timescale of the analysis from sensitivity to rate based information in which the cost of temporally shifting a spike is low to temporal (spike timing) scale where the cost is much higher. For example, if the cost is low (***q*** = 0) this distance equates to a simple comparison among spike counts. For larger values of ***q***, the distance becomes more favorable to deletion or insertion of non-coincident spikes rather than shifting them in time. Thus by adjusting the cost, the metric can be made more sensitive to rate vs. temporal information shared between the two spike trains. Estimates of ***D_v_*** were normalized by the spike rates of each pair of spike trains (***D_v_*** = ***D_v_***/(*n_x_* + *n_y_*), where 0.0 < ***D_v_*** < 1.0; Kreiman et al., 2000). Normally this dissimilarity estimate, ***D_v_***, ranges from 1.0 indicating highly *dissimilar* spike trains to 0.0, in which spike trains are nearly identical. For the purpose of discussion we express ***D_v_*** in terms of *similarity*, ***S_v_***, (where similarity = 1.0 − ***D_v_***) rather than dissimilarity by inverting ***D_v_*** such that ***S_v_*** now ranges from 1.0 (similar) to 0.0 (dissimilar).

**Figure 2:**
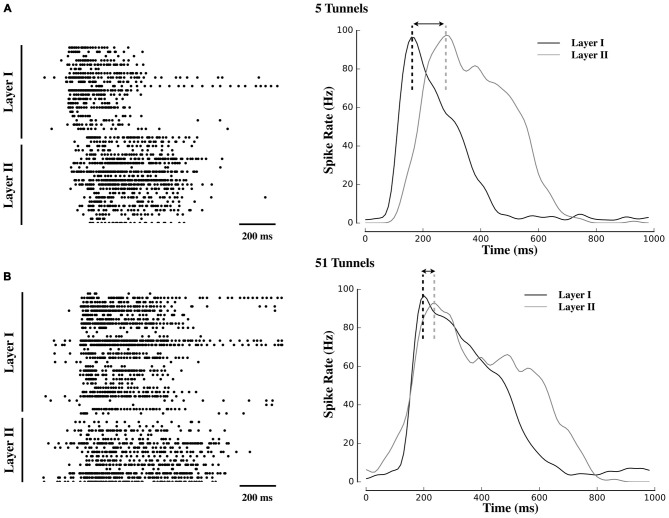
**Delayed propagation of population bursts within decreasing number of tunnels.** Raster plots and associated activity histograms during a propagating burst event from a single microelectrode array (MEA) culture in Group 5T **(A)** and Group 51T **(B)**. Increasing the number of pathways decreased the delay (indicated with arrows) with which spontaneous bursts in Layer I were able to propagate into Layer II.

Spike trains from bursts events that successfully propagated across the tunnels from Layer I to Layer II (criterion > 20% temporal overlap) were collected and spike distance metrics computed over individual events across all possible electrode pairs. Due to the limited number of tunnels connecting each layer, particularly in the 2T and 5T groups, the onset of a burst of activity in Layer I was often followed by a significant delay before a similar event would be initiated in Layer II (compare 51T vs. 2T, Figure 2). Because of the large differences in the propagation delays between a burst in Layer I and Layer II we focused on only those bursts where a burst in Layer I propagated into Layer II and temporally overlapped with a burst in Layer II by at least 20% of the maximum duration of the Layer I burst event. Conversely, fidelity estimates of transmission during the intervals between burst events were defined as the period between the overlapping windows described earlier and therefore included periods in which Layer I or Layer II might be active but not within temporally overlapping burst events.

Our Victor-Purpura estimates were compared with a method similar to a second popular metric reported by van Rossum (2001). In this metric spikes are binned into 1 ms intervals and convolved with an exponential kernel with time constant ***τ***_*R*_ (e.q. 2.1 and 2.2 in van Rossum, 2001). The choice of the exponential kernel was originally motivated in van Rossum’s work by its causal properties and correspondence to the shape of postsynaptic currents. The time constant of the exponential, ***τ***_*R*_, determines the precision with which this distance metric is sensitive. Following convolution with the exponential kernel we then calculated the similarity between the two convolved spike trains as a Pearson correlation to produce a similarity estimate, ***S_R_***, that is bounded from 0 to 1 (dissimilar to similar) that can then be compared directly with Victor-Purpura’s measure producing a similar range of values.

### Statistical Analysis

Analysis of the spike train data was conducted using custom Python (Enthought v1.6.2 64 bit mac distribution; main analysis and plots), C and C + + (Granger causality analysis, Victor-Purpura, Van Rossum), the R statistical package (ANOVA from the ezAnova package, CRAN), Network X network analysis library (v1.9.1) within Python and Gephi for the display of network graphs. CGC was computed using custom C code with GSL, BLAS, and LAPACK libraries by the first author adapted from the Matlab source code freely available from Anil Seth (2010). Unless otherwise noted statistical analysis consisted of two-way ANOVA with group (2T, 5T, 10T, 15T, 51T) and layer (Layer I and Layer II) as factors. This was followed by *post hoc* comparisons using *t*-tests (Python, scipy package). The type I error rate or significance level set at 0.05. The family-wise error rate during multiple *t*-test comparisons was corrected for false discovery rate using Benjamini and Hochberg’s approach Benjamini and Hochberg (1995). Any mean values cited in text represent the mean ± SEM (standard error of the mean).

## Results

### Spontaneous Formation of Network Oscillatory Behavior in Culture

Figure 2 displays raster plots of neural activity for one representative culture from each group. Each raster represents the timing of spontaneous action potentials (spikes) during a 10 min recording from cultures ranging in age from 20–26 DIV. The gray shaded area within each plot corresponds to those electrodes located within the tunnels that separated Layer I (top half of each raster) from Layer II (bottom half). Typical patterns of spontaneous activity consisted of periods of semi-isolated asynchronous spiking scattered across Layer I and Layer II followed by the semi-periodic appearance of intense network-wide bursts. These bursts appear as vertical strips of points in each raster and mirrored in the sharp peaks that appear within the instantaneous firing rates (Population spiking) plotted below each raster. Each raster is accompanied by a 1 s sample of a single burst (right column) to illustrate differences in burst dynamics between Layer I and Layer II and between each group. In these plots, burst events typically began in Layer I with a rapid increase in firing (reflected in the instantaneous firing rates) forming the onset of the burst. In Group 51T this activity was rapidly recruited and initiated firing and a subsequent burst in Layer II before firing in both layers declined. By comparison, in Group 2T there was a noticeable delay between the activation of Layer I and Layer II with intermediate delays in the remaining groups. In fact in some raster plots a double peak appeared in the instantaneous rates (e.g., 10T, 5T, 2T). The presence of this double peak reflects the delay between the initiation of a burst in Layer I and the onset of bursting in Layer II. These delays were particularly pronounced when few tunnels connected the two neural populations and are consistent with extensive delays reported by other laboratories (Baruchi et al., 2008; Tsai et al., 2008; Bisio et al., 2014; Pan et al., 2015).

The mean firing rates among neurons among layers were similar between groups (mean: 0.63 ± 0.31, 0.84 ± 0.20, 0.92 ± 0.16, 1.61 ± 0.51, 1.10 ± 0.13 Hz for Groups 2T, 5T, 10T, 15T, and 51T respectively; *p* > 0.44). Rates did not significantly differ between layers (*p* > 0.28), nor was there a significant interaction between group and layers (*p* > 0.30). Almost half of all neural activity that occurred within each layer did so within the series of spontaneous bursts among all groups and layers (42.6 ± 0.4%, *p* > 0.56) that typify activity patterns among cultured cortical (e.g., Pasquale et al., 2008; Downes et al., 2012; Gritsun et al., 2012) and hippocampal (e.g., Maccione et al., 2010; Brewer et al., 2013; Pu et al., 2013) neural networks.

In this study we used timed-sequential plating to promote primarily feed-forward connectivity from Layer I to Layer II (see "Functional Connectivity Between Layers" Section below). To propagate from layer to layer bursts that originate in Layer I must recruit neurons to fire in Layer II to ignite a burst of activity within Layer II. Once that burst has been initiated in Layer II the activity in Layer II likely represents the contribution of neurons from within this layer and any additional driving influence over activity produced during transmission by neurons within Layer I upon neurons in Layer II. The presence of this additional driving influence from Layer I into Layer II could appear in the dynamics of Layer II in a number of ways. For example, this additional driving input might appear as an increase in firing rates among neurons in Layer II or may extend the duration of burst events occurring in Layer II; these effects should increase with increasing number of tunnels.

In fact, the number of tunnels did result in significant increases among a number of measures of spike dynamics in Layer II. For example, while the number of tunnels did not have a significant effect on the overall rate of bursting in each group overall (*p* > 0.7), the rate of bursting did appear to be slightly higher in Layer I compared to Layer II (1.6 ± 0.4 vs. 1.0 ± 0.2 bursts per minute) in 19 of 24 cultures but this difference was not significant (*p* > 0.762). Increasing the number of tunnels did result in a significant increase in the duration of bursts occurring in Layer II (578.3 ± 38.7 ms) relative to Layer I (489.3 ± 28.1 ms; *p* < 0.002). There was also no significant interaction among burst durations between tunnels and layer (*p* > 0.28). An increase in the number of tunnels resulted in an increased average rate of firing *during* each burst (42.8 ± 7.9 Hz, 36.0 ± 4.5 Hz, 46.6 ± 4.4 Hz, 49.1 ± 11.4 Hz, and 54.1 ± 4.9 Hz for Groups 2T to 51T, respectively, *p* < 0.016) and an increased peak firing rate within bursts (79.1 ± 8.6 Hz, 82.4 ± 7.8 Hz, 107.1 ± 10.1 Hz, 101.9 ± 10.7 Hz, and 112.5 ± 8.6 Hz, for Groups 2T to 51T, respectively, *p* < 0.047) with no significant differences between layers for either measure (*p*’s > 0.096).

In our earlier report (Pan et al., 2015) we provided evidence for significant delays between bursts in Layer I and Layer II when activity was electrically evoked in a two-chamber system containing cortical populations like those used here. In that study an electrode in Layer I was stimulated periodically and evoked a burst of activity within that layer. We observed a significant increase in the likelihood and significant decrease in the delay in which a burst would propagate from Layer I to Layer II with increasing number of tunnels connecting each layer. In this study we observed similar increases. The likelihood of a spontaneous burst that had originated in Layer I to propagate into Layer II was lowest in Group 2T (0.22 ± 0.22) and became significantly more likely in 5T (0.57 ± 0.9), 10T (0.44 ± 0.9), 15T (0.43 ± 0.5), which where equivalent to each other, relative to 51T (0.72 ± 0.11) which produced the highest probability of successful propagation, *p*’s < 0.5. In fact, only one of four cultures in the group with two tunnels (Group 2T) produced spontaneous bursts in Layer I that were able to propagate to Layer II.

An increase in the number of tunnels was also associated with a significant *decrease* in the delay between the onset of bursts in Layer I and subsequent bursts in Layer II (e.g., compare raster plots and burst histograms between 51T and 5T in Figure 2). To quantify this delay we estimated the latency between the point at which peak neuronal firing occurred within a burst in Layer I, and time of peak firing in Layer II (illustrated as a dashed black or gray vertical line in Figure 2). Electrically evoked propagation in our earlier report (Pan et al., 2015) indicated the presence of a significant increase in peak-to-peak delays in Layer I and Layer II. Here we observed similar delays present among spontaneous activity and its propagation between layers with delays between the onset of a burst in Layer I to a burst in Layer II of 90 ± 21 ms, 147 ± 20.ms, 389 ± 55 ms, 172 ± 24 ms, 280 ± 69 ms in Groups 51T, 15T, 10T, 5T, 2T, respectively (*p* < 0.05). If these delays were due simply to axonal conduction velocity between neurons in Layer I to Layer II alone they should not only be similar across groups but also limited to delays less than approximately 7 ms, assuming a conduction velocity of 0.4 ms (Patolsky et al., 2006). The substantially longer delays we observed in this study are likely indicative of properties of burst propagation rather than axonal conduction velocity alone.

### Functional Connectivity Between Layers

Neurons simultaneously plated into two chambers and allowed to interconnect naturally develop a bias in the timing and direction of propagation from one chamber to the next in a master-slave like topology (Baruchi et al., 2008; Bisio et al., 2014). In this study we experimentally controlled the direction of propagation through timed sequential plating from Layer I to Layer II to ensure predominately feed-forward connectivity from Layer I to Layer II (Pan et al., 2011, 2015). To verify the bias in directionality (Pan et al., 2011) and quantify the degree to which networks were feed-forward, feed-back, or bi-directional, we focused on the timing information contained within the axonal propagation delays recorded by electrode pairs located along a small subset of tunnels (e.g., Figure 1D). Electrodes within the tunnels provided a unique opportunity to directly measure and assess a subset of communication (spiking) traveling between each layer (Note: not all tunnels contained electrodes however). Each pair of electrodes along a tunnel were spaced 200 μm apart and because of the confined recording space within a tunnel (3 × 10 × 400 μm, height × width × length), resulted in extraordinarily high magnitude extracellular signals near 1 mV recorded on electrodes within each tunnel (typical signals range from 10 to 100 μV within the layers; Pan et al., 2011; Wang et al., 2012). Based on spike train data from each electrode pair we computed a traditional cross-correlation metric (CC) and compared the results with more recent conditional Granger causal measures (Cadotte et al., 2008; Natarajan et al., 2013; Pan et al., 2015).

**Figure 3:**
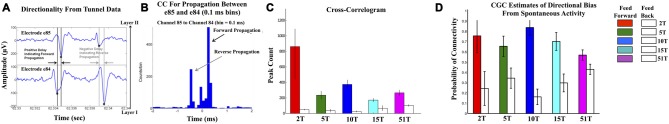
**Confirmation of Feed-Forward functional connectivity between Layers I and II. (A,B)** illustrate the computation of correlation metric (CC) in which the relative timing of spikes measured from electrode pairs (top and bottom of **(A)**). With an electrode separation of 200 μm and expected conduction velocities between 0.2 and 0.8 m/s (Patolsky et al., 2006), any significant peaks should appear at delays between 0.2 and 0.8 ms. In each CC spikes were accumulated in 0.1 ms bins in a window ±2 ms surrounding each spike in the reference train. **(B)** shows an example of the CC computed from the electrode pair 84 and 85 (column 8 and row 4) located along a tunnel in the 51T group. Forward propagation manifests as a peak in CC at positive time lags that is higher than any peaks in the reverse direction. Peaks and inferred direction were computed for each electrode-tunnel-pair in the 2T, 5T, 10T, 15T, and 51T groups and proportion of feed-forward vs. feedback connections calculated. **(C)** plots the results of that calculation in terms of normalized frequency of feed-forward (colored bars) vs. feedback connectivity for each group. Feed-forward connections were observed significantly more often than feedback in each group. We also computed the conditional Granger causality (CGC) metric on spike train data. **(D)** The results of our CGC analysis of spontaneous were consistent with those from CC with the majority of connectivity extending from neurons in Layer I into Layer II through the tunnels with low levels of feed-back.

Figures 3A–C show the results of the CC analysis in which the majority of CCs contained peaks at positive time lags indicative of propagation from neurons in the input layer (Layer I) through axons in tunnels to neurons within the Layer II. Average conduction velocity estimates based on the lag-time to the peak CC (Figure 3B) was approximately 0.6 ms, consistent with values we have estimated earlier within the tunnels (Dworak and Wheeler, 2009; Pan et al., 2011; Brewer et al., 2013) and are consistent with estimates provided by other methodologies in other laboratories (e.g., Patolsky et al., 2006). Figure 3C shows the mean peak CC values in the feed-forward Layer I to II for each group relative to feedback (adjacent white bars) from Layer II to I. Peaks in the CC results from the tunnels were significantly higher in the feed-forward compared to a feedback direction (average counts per bin: 321 ± 53 vs. 54 ± 11, *p* < 0.005) consistent with primarily feed-forward directionality. Figure 3D displays the results based on the conditional Granger causal analysis (CGC) estimated from tunnel activity and is depicted as the probability of observing a significant connection in a feed-forward (colored bars) vs. feedback (white bars) direction. Like the CC results, CGC estimates of causal strength of connectivity within tunnels were significantly higher among feed-forward vs. feedback connectivity (overall mean 0.71 ± 0.01 for feed-forward vs. 0.29 ± 0.01 for feed-back, *p* < 0.0005).

**Figure 4:**
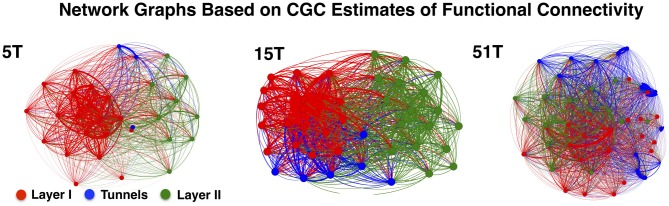
**Network graphs depicting functional connectivity based on CGC causal strength.** Network graphs of three representative cultures from Groups 5T, 15T, and 51T. Each node denotes an electrode and each is colored according to the electrodes original location (Red denotes electrodes in Layer I, Green in Layer II while Blue for Tunnel data). The network layout was computed using a spring-mass (Force-directed) mapping to illustrate the difference in the functional connectivity between the coupled networks in each group. The numbers of tunnels between layers play a vital role in the modularity of the network (i.e., the number of connections a node in a single layer has with other nodes within across the layers) producing unique clusters representing each layer. These clusters begin to merge as the number of tunnels increase permitting greater connectivity and coupling between layers.

Figure 4 illustrates the functional connectivity derived from the CGC analysis in the form of network connectivity graphs for representative cultures in Group 5T, 15T, and 51T. Each graph presents a force-directed spring-layout (Kamada and Kawai, 1989) for representative cultures in 5T, 15T, and 51T to visually illustrate some of the primary differences between the network topologies that resulted from changes in the number of tunnels. In a force-directed layout topology, the positions of nodes (electrodes) are located according to the strength of connection between nodes (edges) with strength represented as a mechanical force of a spring that pulls upon other nodes. Each network edge corresponds to the presence of a significant functional connection detected using CGC causal estimates (only links where *p* < 0.0001 were included in our analysis). In this representation nodes that are strongly connected will tend to draw together. Layer I is shown in red, Tunnels in blue, and Layer II in green. First, nodes tend to cluster by layer reflecting the higher density of connections within each layer compared to those cross layers. Second, nodes located within the tunnels typically cluster near Layer I rather than Layer II perhaps reflecting the stronger links from Layer I to the tunnels relative to those from the Tunnels to Layer II. However, increasing the number of tunnels produced progressively more Granger causal links between Layer I and Layer II, which manifests in the spring-plots as a gradual merging of layers (compare 5T to 51T). An analysis of the average number of connections per neuron, known as node degree within network connectivity analysis (Boccaletti et al., 2006), indicated a significant increase in the average node degree from a low of 27.4 ± 0.7 in 2T to a high of 42.2 ± 0.7 connections per node in Group 51T group overall but no significant differences between layers in any group. The increase in node degree by number of tunnels was also true for the average “in” degree (average number of incoming connections, 26.06 ± 0.66 in 2T to 40.0 ± 0.9 in 51T) and “out” degree (26.0 ± 0.7 in 2T to 40.1 ± 0.9 in 51T) per node (neuron). Finally, the presence of recurrent or reciprocal connections is well known in cortex (e.g., Holmgren et al., 2003; Song et al., 2005), often seen between brain areas (e.g., Song et al., 2011), and are known to have a profound influence of network dynamics (e.g., Tononi and Sporns, 2003). Though relatively rare at 10.6 ± 0.8% of all connections across groups, we found that the average percentage of reciprocal connections did change with the number of tunnels with the highest prevalence of 14.6 ± 1.9% in group 2T and decreasing to a low of 5.8 ± 0.9 in Group 51T, *p* < 0.01. Comparison among layers indicated a consistently higher prevalence of reciprocal connections in Layer I compared to Layer II in each group, average 162.5 ± 9.8 vs. 110.8 ± 10.6.

### Spike Train Similarity Metrics and Nature of the Neural Code During Transmission Across Layers

To determine the fidelity of transmission of spike trains between Layer I and Layer II we compared the similarity of spike trains during burst events that propagated from Layer I to Layer II when those events overlapped in time by at least 20% or more (see “Materials and Methods” Section). The percent temporal overlap among bursts that originate in Layer I and propagate into Layer II increased with increasing number of tunnels. The average percent overlap was lowest in Group 2T at 54.9 ± 12.3% and increased with number of tunnels from 76.6 ± 2.4% in 5T, 82.4 ± 7.2% in 10T, 83.2 ± 3.8% in 15T, and was highest in Group 51T at 94.3 ± 0.6% temporal overlap (*p*’s < 0.05).

**Figure 5:**
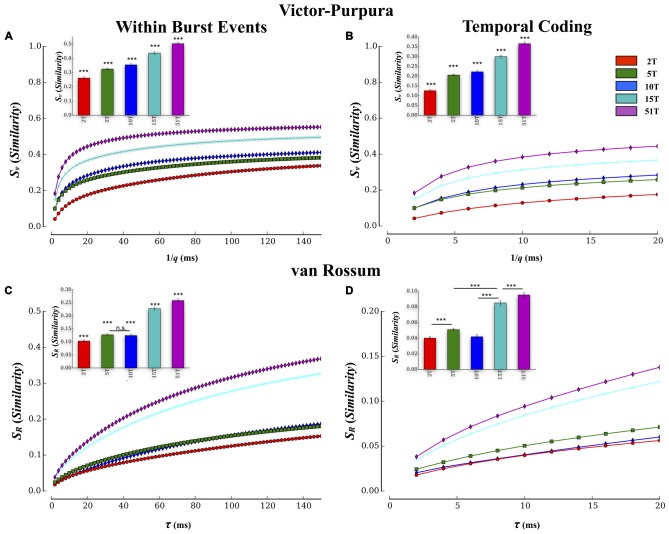
**Estimates of transmission fidelity based on the Victor-Purpura and Van Rossum’s metric.** Similarity estimates among spike trains provided by the Victor-Purpura and van Rossum’s metric were averaged over electrode pairs for each MEA culture producing a composite score at each level of the metric’s timescale parameter value (1/***q*** and ***τ***_R_). Associated group means are shown as bar plots (inset). Increasing the number of tunnels resulted in a greater transmission fidelity between layers based on similarity estimates using Victor’s **(A,B)** and van Rossum’s metric **(C,D)**. Increased fidelity was apparent at both rate (1/***q*** > 80 ms and ***τ***_R_ > 80 ms) and temporal scales (1/***q*** < 20 ms and ***τ***_R_ < 20 ms) of each measure. These estimates were conducted during periods in which bursting was actively propagating and temporally overlapped from Layer I and II. Similar results were also observed if we computed these metrics to include the entire duration of each event (i.e., start of burst in Layer I to end of burst in Layer II; ***p < 0.001).

**Figure 6:**
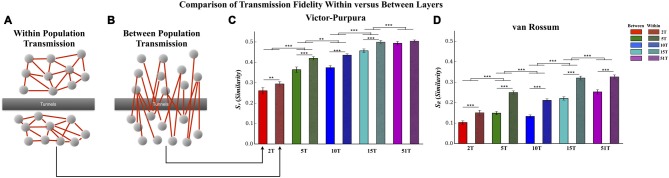
**Comparison of transmission fidelity within vs. between layers.** Unlike transmission between layers where activity among one population of neurons must cross the tunnels to reach the second layer (illustrated in **B**), transmission within each layer (illustrated in **A**) would be unimpeded tunnels, should represent intrinsic wiring of the network, and might therefore produce the highest possible fidelity estimates during transmission. In fact in the majority of cases transmission fidelity was higher during measurements of within layer communication compared to communication between layers based fidelity estimates using the Victor-Purpura **(C)** and von Rossum metrics **(D)**. However, we also found that increasing the number of tunnels led to an overall increase in fidelity even among the communication within each layer. This latter effect is surprising in that within layer connectivity should have been the same across the groups (Error bars represent 95% confidence intervals; **p < 0.01, ***p < 0.001).

Pairwise results were then averaged within each culture to form a composite similarity score that was then compared between each group at each temporal resolution (i.e., cost), ***q***, shown in Figure 5A. The overall transmission fidelity based on the average of the Victor-Purpura similarity measure, ***S_v_***, is also included as a bar graph (inset) for convenience in Figure 5A. In this study increasing the number of tunnels that connect two small populations of neurons significantly increased the fidelity with which they communicated spike trains from neurons in Layer I to neurons in Layer II (*p*’s < 0.001). Within each group, rate-coding (i.e., 1/***q*** > 50 ms) produced the highest similarity scores compared to finer temporal scales (1/***q*** < 20 ms), which is merely a reflection of the fact that this function is mathematically monotonically increasing with 1/***q***. The interesting comparisons are the between group comparisons at temporal scales and rate based scales. Comparisons at these two scales indicated the presence of significant between group differences while increasing tunnels indicative of the presence of both rate and temporal information present within the spike trains. Average transmission fidelity estimates at cost parameters associated with a course rate modulation base code (1/***q*** > 50 ms) was significantly higher in 51T compared to 15T, 10T, 5T, and 2T groups (means: 0.53 ± 0.01, 0.49 ± 0.01, 0.39 ± 0.01, 0.36 ± 0.01, 0.30 ± 0.01 (*p*’s < 0.038). The region of the plot in Figure 5A associated with finer temporal coding fidelity estimates (1/***q*** < 20 ms) is shown in Figure 6B (and average fidelity, inset) and depict significantly higher fidelity with increasing tunnels at temporal scales (*p*’s < 0.001). We also obtained similar results (not shown) when we instead estimated fidelity during the entire duration of each bursting episode (from the very start of a burst event in Layer I to the termination of bursting in both Layer I and Layer II rather than during the overlap alone) and also after temporally aligning (time shifting) individual burst events in each layer by their respective peak firing times during each event before computing our fidelity metrics.

#### Comparison of Results with van Rossum’s Metric

Spike train similarity estimates produced by van Rossum’s metric are plotted in Figure 5C and temporal region expanded in Figure 5D. Unlike the edit distance based metric of Victor-Purpura, van Rossum’s metric computes the correlation among spike trains whose scale, ***τ***_*R*_, represents the shape and integration constant of synaptic currents. Although van Rossum’s metric tended to produce lower overall estimates of similarity than Victor-Purpura’s cost base metric (compare average values shown in Figure 5A inset vs. Figure 5C inset) estimates of fidelity from this metric were consistent with that from Victor-Purpura. Like the results from the Victor-Purpura measure, the overall similarity of spike trains during transmission increased with increasing number of communication pathways (tunnels; *p*’s < 0.004; Figures 5B,D). Average similarity estimates at scales corresponding to a rate modulation based code (***τ***_*R*_ > 50 ms) were significantly higher in 51T compared to 15T, 10T, 5T, and 2T groups (means: 0.12 ± 0.01, 0.18 ± 0.01, 0.16 ± 0.01, 0.27 ± 0.04, 0.30 ± 0.01, *p*’s < 0.006). Similarity among transmitted spike trains at temporal scales (***τ***_*R*_ < 20 ms) also increased with increasing number of tunnels (Figure 5D, *p*’s < 0.009) and like the results based on Victor-Purpura’s measure, provide additional support for the presence of temporal information contained within spike trains whose fidelity increased with increasing number of tunnels.

#### Transmission Fidelity Within vs. Between Layers

**Figure 7:**
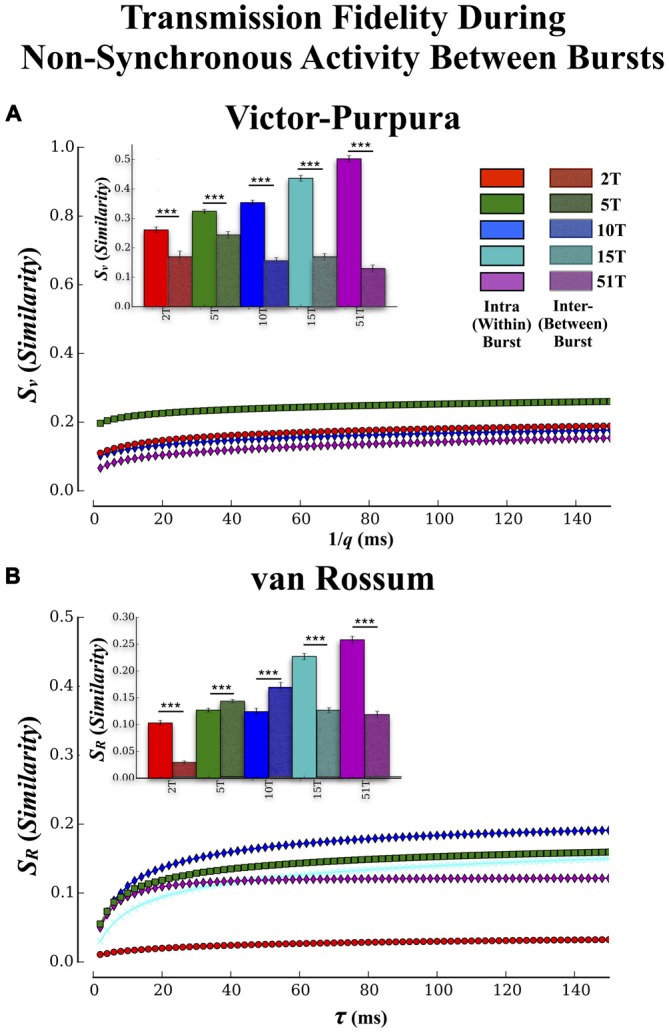
**Transmission fidelity during synchronous population bursting vs. asynchronous single unit activity outside bursts.** The role synchrony may play during transmission of information among neural populations is still a matter of debate. Almost half of all activity we observed in these cultures occurred during the intervals between population wide bursts of activity. If synchronized bursting promotes information transmission then fidelity should be much higher during transmission within the bursts compared to transmission during the intervals between the bursts. **(A,B)** show the results of our fidelity estimates based on Victor’s and van Rossums metric, respectively for activity that occurred *between* bursting compared to *within* bursting (repeated from Figures 5A,B). Transmission fidelity was significantly degraded when it occurred in the absence of synchronous firing outside of bursts relative to inside burst events (Error bars represent 95% confidence intervals; ***p < 0.001).

In this study increasing the number of tunnels that connect two neural populations resulted in significantly higher transmission fidelity of spike trains between Layer I and Layer II. By comparison transmission among neurons within each layer (illustrated in Figure 7A) should be unimpeded by any tunnels whatsoever. This fact suggests that fidelity should be highest among neurons communicating within each layer compared to between layers (Figure 7B) whose fidelity we reported earlier (cf. Figure 5). Figure 6 directly compares the average transmission fidelity between layers (repeated from Figures 5A,C, insets) vs. fidelity estimates among neurons within Layer I and Layer II for the Victor-Purpura (Figure 6C) and van Rossum metrics (Figure 6D). In groups 2T, 5T, 10T, and 15T, within layer communication fidelity was significantly higher than that between layers reflecting the impact the absence of any barrier (i.e., no tunnels) had upon communication. Moreover the advantage of within compared to between layer fidelity decreased with increasing number of tunnels reflecting the increased level of connectivity provided by the 51 tunnels. The greatest change in fidelity occurred among networks in the 5T and 10T groups compared to 2T and 15T groups with smallest change from within to between layer transmission fidelity observed in 51T (*p*’s < 0.001) where there was now no significant difference. Finally, because there are no tunnels to restrict communication among neurons within each layer and since this is true in each group regardless of the number of tunnels that connect each layer, changing the number of tunnels should have little effect on the magnitude of fidelity estimates within each layer when compared across groups. However, this was not the case. Surprisingly, by increasing the number of tunnels we not only enhanced between layer communications (Figure 6B) but was also appeared to enhance within layer communication fidelity (Figure 6A). The average within layer fidelity estimates significantly increased from 0.28 ± 0.01 in 2T, 0.40 ± 0.01 in 5T, 0.41 ± 0.01 in 10T, 0.48 ± 0.01 in 15T, and 0.50 ± 0.01 in 51T (*p*’s < 0.01). It appears that increasing the number of tunnels improved the fidelity of communication among neurons whether fidelity was measured between layers or within each layer.

#### Is Population Synchrony During Transmission Important for High-Fidelity Communications?

There have been suggestions that bursting may be detrimental to rate based coding by limiting the bandwidth with which firing can vary during these events while others suggest bursting may be crucial for successful transmission by enhancing post-synaptic firing probabilities via coherent firing (e.g., Fries, 2005, 2009). To determine if synchronous bursting promoted communication fidelity we compared our earlier results (cf. Figure 5) that focused on transmission during the bursts with an analysis of spike trains measured between each burst event (i.e., non-bursting periods within intervals between burst events). The Victor-Purpura and van Rossum estimates are shown in Figures 7A,B, respectively. The similarity between spike trains in each layer was much lower when measured outside of burst relative to inside burst events in each group (Figure 7A inset) and there were now no significant differences between groups (*p* > 0.63). Similar trends were observed using fidelity estimates based on van Rossum’s measure in Figure 7B, (inset) and clearly support the role of synchronous activity (bursting) provides enhancing communication between the two populations of neurons located in Layer I and Layer II, respectively.

## Discussion

Recent theories of neuronal coding emphasize the role of synchronicity in the encoding and transmission of information within the brain (e.g., Fries, 2005, 2015). However, much of our understanding of how that information is transmitted among neuronal assemblies or the properties during that transmission (e.g., neural code) have been derived from computational models that make simplifying assumptions that may influence the nature and properties of any neural code that is expressed. While *in vivo* investigations may be difficult because of limited access, *in vitro* methods offer an unparalleled view into detailed multi-scale information about neuronal activity and the functional connectivity over which that activity occurs. In this article we describe the creation of an *in vitro* analog of a classic feed-forward architecture between two small cortical populations that represent the layers of the architecture. Each population/layer was connected by a series of micro-scale tunnels that provide communication pathways via axonal projections between each layer. We then systematically manipulated the number of pathways (tunnels) to determine whether increasing number of communication pathways provided by the increase number of tunnels would: (1) modify the dynamics of spontaneous neural activity including synchronous bursting and burst propagation between layers; and (2) predict that more communication pathways would result in higher transmission fidelity of information embedded within spike trains among neurons communicating across each layer.

### Improved Transmission Fidelity with Increasing Number of Tunnels

Increasing the number of tunnels and hence, communication pathways, did lead to an enhanced transmission fidelity measured among spike trains transmitted between each layer based on our two similarity metrics (cf. Figure 5). In each group we observed significant increases in fidelity for both rate based and temporal scales. Propagation of activity in feed-forward architectures has been explored extensively in the modeling literature (Diesmann et al., 1999; Câteau and Fukai, 2001; Gewaltig et al., 2001; Kistler and Gerstner, 2002; van Rossum et al., 2002; Litvak et al., 2003; Vogels and Abbott, 2005; Kumar et al., 2008, 2010; Brette, 2012; Renart and van Rossum, 2012) and recently within an *in vitro* acute cortical slice (Reyes, 2003) and dissociated neural culture (Pan et al., 2011, 2015; Vincent et al., 2012; Natarajan et al., 2013). It has sometimes been suggested that the formation of synchronized correlated activity (bursting) may be *detrimental* to the expression of a rate modulated neural code (Shadlen and Newsome, 1998; Mazurek and Shadlen, 2002; Litvak et al., 2003; Mehring et al., 2003; Kumar et al., 2008). This viewpoint contrasts with a number of recent hypotheses emphasizing the need for feed-forward synchronous firing to promote encoding and increase reliability of transmission (Fries, 2009; Ainsworth et al., 2012; Nikolić et al., 2013; Ratté et al., 2013). When we compared our results estimated during the highly synchronous firing among burst events with those from the relatively non-synchronous low rate of firing outside of these burst events we found that although there was some evidence for a rate based code, the fidelity of transmission was significantly poorer than that measured during bursts. In fact, increasing the number of tunnels had little effect on the fidelity of communication between layers (Figures 7A,B) during transmission outside synchronous bursting. Together, these results provide further support for the importance of synchronized activity for the successful transmission of information among neural populations and the role the number of communication pathways can have on the fidelity of those transmissions.

### Within vs. Between Layer Communication

Increasing the number of tunnels and presumably the number of axonal projections from Layer I to Layer II enhanced the fidelity of transmission of spike trains across each layer. Though enhanced, how do these levels of fidelity compare with communication within those layers? After all, communication within each layer should be unimpeded by any tunnels and should therefore produce the highest levels of fidelity overall. It is perhaps not surprising then, that when we compared the fidelity during transmission between layers with that during communication among neurons within each layer we found that the fidelity within each layer was in fact significantly higher than between layers. What is surprising however is that increasing the number of tunnels not only increased the communication fidelity between layers but also increased the fidelity *within* each layer (cf. Figure 6). For example, one prediction might be that while increasing the number of tunnels would increase the fidelity between layers, connectivity within each layer would be unaffected and hence, no effect upon transmission within layers should occur. Instead, it appears that increasing the number of tunnels increased not only the fidelity of transmission between layers, but also increased the transmission fidelity within each layer. In essence, it appears as if spiking across both neural populations in Layer I and II became increasing similar to each other as the number of axonal projections connecting each population increased from the tunnels. This might be expected in Layer II which was partially being driven by activity in Layer I due to the predominate feed-forward connectivity we showed in Figure 3, but why would the similarities in Layer I increase as well? A number of observations may provide important clues.

First, the overall increase in fidelity with increasing number of tunnels is not likely to be due simply to the fact that networks are slightly older in Layer I than Layer II (recall our timed sequential plating method establishes feed-forward connectivity through delayed sequential plating). In this experiment all groups were subject to the same timed-plating procedure.

Another possibility is that the overall number of connections among neurons in each population increased in parallel with the increase in number of tunnels. After all, the presence of feed-forward connectivity at minimum would imply a bias for an increased number of post-synaptic connections in Layer II, a number that should only increase with more tunnels. In fact we did find that the average number of connections (i.e., node degree) increased with more tunnels. If true, perhaps the increase in the number of connections may have then contributed to the increased similarity among spiking within each of the neural populations.

Neurons with higher node degrees (greater number of connections) have also been associated with the formation of rich-club networks and “hub” neurons implicated in governing neural dynamics and modulating the dynamical interactions among other lower-degree nodes (Crossley et al., 2013; Senden et al., 2014). The hub neurons have also been associated with enhanced network communication and information transfer (Sporns et al., 2007; de Reus and van den Heuvel, 2013; van den Heuvel and Sporns, 2013), and hypothesized to serve as a network backbone for transmission and integration of information in the brain (van den Heuvel et al., 2012; Towlson et al., 2013; Mišić et al., 2014; de Reus and van den Heuvel, 2014) *in vitro* (Yu et al., 2008; Shimono and Beggs, 2015; Timme et al., 2014; Schroeter et al., 2015).

### Effect on Dynamics, Potential Mechanisms, and Relationship to Wave Propagation *In Vivo*

Increasing the number of tunnels that connect each layer also led to substantial increases in the likelihood with which bursts are able to propagate from Layer I to Layer II. This result is consistent with our prior report based on evoked activity (Pan et al., 2015). Increasing the number of tunnels also resulted in significant delays between the propagation of a spontaneous burst in Layer I and subsequent propagation into Layer II (cf. Figure 2 51T vs. 2T), which is also consistent with the delays we reported earlier (Pan et al., 2015), known to vary with culture maturity in a tunnel (gap) system similar to ours (Baruchi et al., 2008; Bisio et al., 2014), and observed by others during direct chemical manipulation of synaptic transmission (Tsai et al., 2008) or natural degree of connectivity (Shein Idelson et al., 2010) within various patterned topologies. These delays can be substantial, sometimes as long as several 100 ms and are not likely to be the result of simple delays induced by axonal conduction velocities as those velocities are much faster and would produce substantially shorter delays than those we report here. In our earlier report we showed using our CGC measure that increasing the number of tunnels led to stronger connection strengths on average between each layer which in turn, may have led to stronger overall coupling between the two cortical populations separated by the tunnels (Pan et al., 2015). Perhaps this stronger coupling enabled by the increased number of projections between each population provided a stronger driving force from Layer I to Layer II. This should have lead to better synchronization between the activity in each layer and could explain both the increasing likelihood and decreasing propagation delays while at the same time enabling better overall communication. In fact a recent and popular view of information transmission known as the “communication-through-coherence” highlights the important synchronicity both within and between populations of neurons that communicate with each other in the brain (Fries, 2005, 2015). According to this view, coherently oscillating neuronal groups provide the basis for effective communication. This synchronicity or neural coherence in firing between populations may also offer other advantages. For example, the transient emergence of coherent activity may bind neural activity evoked by a stimulus across different brain areas into one perceptual object, a concept known as the binding-by-synchronization hypothesis (von der Malsburg and Schneider, 1986; Singer and Gray, 1995; Engel et al., 1997, 2001; Singer, 1999). This coherence may also represent a basic mechanism with which information is routed within the brain (Fries et al., 2002). However, there is little detailed knowledge at the cellular to network level correspondence concerning the properties of communication among brain areas.

At the micro-level, we know from the MEA literature that the identities of the neuron or neurons that are activated early during the initiation of the burst can have a profound influence on whether a burst will then actually occur (Maeda et al., 1995; Yvon et al., 2005; Eytan and Marom, 2006; Eckmann et al., 2007, 2008; Cohen et al., 2008; Ham et al., 2008; Pan et al., 2009; Orlandi et al., 2013), and can also determine the subsequent pattern of firing that then follows during the burst event itself (Eckmann et al., 2008). For example, Eytan and Marom (2006) showed that particular neurons among a select set of neurons (which they call “leader” neurons) tended to precede individual bursts. The activity of this pool of leader neurons, typically composed of approximately five neurons, persisted over many hours. Which leader neuron appeared during this initiation period was also predictive of the pattern of spontaneous activity that followed. They also showed that, although a burst appears to be an all-or-nothing threshold-governed event, increasing the number of neurons and hence, action potentials that participate *during* *the initiation* of a burst *decreases* the amount of time needed to reach synchrony within that burst. In the case of our two-chamber system, the increase in the number of tunnels and presumably number of projections from neurons in Layer I to Layer II might therefore be expected to decrease the amount of time needed to reach synchrony accordingly and may explain why more tunnels decreased this latency tremendously.

Eytan and Marom (2006) also found that which neuron from Chamber A provided input into chamber determined the latency to a subsequent burst in Chamber B. In a clever experiment, Eytan and Marom electrically coupled two independent cortical cultures, labeled X and Y, using a stimulus generator. In this experiment stimulation pulses at a location in Y (50 μA, 400 ms bi-polar pulse) were explicitly timed with spiking on select electrodes in X (i.e., neural activity in X drives Y). Since, each culture is independent there would be little reason therefore, to believe that the choice of which neuron from culture X could have any influence upon the delay to a corresponding burst in Y. On the contrary, they showed that when the neuron selected from X to provide input into Y was a member of the pool of putative “leader” neurons mentioned earlier, that neuron was far more efficient at eliciting a burst and doing so faster in Y than other neurons. They argued that when a strongly connected neuron among culture X such as a leader neuron is read by Y, bursts in Y appear almost simultaneously with, or in some cases actually slightly temporally precede bursts in X. If true, this might suggest that one of the effects of reducing the number of tunnels that connect each layer may be to decreasing the likelihood that the neural population in Layer II receives stimulation from one of these leader neurons in Layer I. This factor, in conjunction with a generally higher degree of coupling between layers with increased tunnel number may explain both the likelihood of propagation and apparent delays we observed.

### A New Tool with Which to Study Information Transmission Among Neural Populations

Finally, we comment that the two layer neural system we employ here may provide a useful new *in vitro* model with which to conduct detailed study of properties of propagation *in vivo*. At the macro scale, sensory processing, cognition, and motor control in the brain dynamically engage select neural populations. During these activations the activity of select populations may remain localized in space and time to a particular area or may propagate as a wave between distinct and perhaps remote neural populations and may represent a natural mode of information propagation (Ermentrout and Kleinfeld, 2001; Rubino et al., 2006). These wave fronts are composed of brief bursts of spikes that sweep across the network (e.g., Keane and Gong, 2015; Townsend et al., 2015). In fact the mechanisms governing these propagating waves have become a topic of great interest. Potential mechanisms that influence this phenomenon include cellular and synaptic properties and of course the structure of connectivity such as the distribution of connection lengths (i.e., ratio of short and long-range connections as in small-world connectivity; see Wang, 2010, for a review) or amount of synaptic connectivity (e.g., Ermentrout, 1998; Golomb and Ermentrout, 1999) perhaps reminiscent of that we explicitly attempted to manipulate with the tunnels. In fact, recent years have witnessed a surge in interest of investigating the relationship between structural and oscillatory dynamics and its potential role in neurophysiological disorders including schizophrenia (e.g., Liu et al., 2008b; Lynall et al., 2010) in which functional *dysconnectivity* is thought to play a significant role (Stephan et al., 2009; Phillips and Uhlhaas, 2015), autism (Uhlhaas and Singer, 2006; Rippon et al., 2007), or other neurological diseases (He et al., 2007, 2009). This rather simple multichamber system may provide an ideal means with which to directly manipulate connectivity between neural populations under a variety of conditions and do so with unparalleled access.

## Summary and Conclusions

In this study we have also shown that both rate and temporal based information appear to be transmitted across at least one layer. A variety of factors are thought to influence information transmission including the balance of excitation and inhibition, functional connectivity, and intrinsic properties of the neurons themselves (Shadlen and Newsome, 1994). It is not clear however, how the fidelity of these transmissions may be affected, or what coding (rate vs. temporal) survives when this activity is propagated across multiple layers rather than the two studied here. On this point the modeling literature diverges significantly. Early reports suggested that only rate information could successfully propagate through multiple layers (van Rossum et al., 2002; Vogels and Abbott, 2005) while others reported that under certain conditions temporal information could propagate (Aertsen et al., 1996; Diesmann et al., 1999; Gewaltig et al., 2001; Litvak et al., 2003; Kumar et al., 2008) or rapidly decay after transmission across only just a few layers (e.g., Shadlen and Newsome, 1994). We have recently created and are now conducting measurements of activity propagating across up to a four layers and it will be interesting to see how these different factors interact under these conditions.

Finally, the combination of structured connectivity provided by these PDMS devices with state of the art large-scale histological and electrophysiological measurement provides an attractive new platform that can be adapted to a number of current problems. For example, the degree of convergence among pathways, transmission in the presence of feed-back where connectivity loops back onto the original source layer, to explore the role of noise during the reading or writing of neural codes (Stanley, 2013), or as a tool to mimic *in vivo* connectivity (e.g., the trisynaptic loop in the hippocampus) exploring the properties of information encoding and transmission in an *in vitro* analog of *in vivo* architectures.

## Author Contributions

LP and SA collected the data. TBD and SA conducted the analysis. TBD, BCW, SA and GJB wrote the article.

## Funding

This work was supported in part by the National Institutes of Health research grant NS 052233.

## Conflict of Interest Statement

The authors declare that the research was conducted in the absence of any commercial or financial relationships that could be construed as a potential conflict of interest.
